# Costs of Extrapulmonary Nontuberculous Mycobacteria Disease, Denmark, 2005–2017

**DOI:** 10.3201/eid3203.251548

**Published:** 2026-03

**Authors:** Victor Naestholt Dahl, Andreas Arnholdt Pedersen, Michael Høffding Ibsen, Ole Hilberg, Anders Løkke, Andreas Fløe

**Affiliations:** Aarhus University Hospital, Aarhus, Denmark (V.N. Dahl, A. Fløe); Odense University Hospital, Odense, Denmark (A.A. Pedersen); University of Southern Denmark, Odense (A.A. Pedersen, O. Hilberg, A. Løkke); i2minds, Aarhus (M.H. Ibsen); Lillebaelt Hospital, Vejle, Denmark (M.H. Ibsen, O. Hilberg, A. Løkke)

**Keywords:** bacteria, tuberculosis and other mycobacteria, respiratory infections, nontuberculous mycobacteria, extrapulmonary, healthcare economics, cost analysis, public health, psychosocial support, Denmark

## Abstract

We estimated the direct and indirect costs associated with extrapulmonary nontuberculous mycobacteria (ENTM) disease in Denmark during 2005–2017. ENTM disease was associated with substantially higher healthcare costs, lower employment income, and increased public benefits before, around, and after diagnosis. Our findings highlight the substantial socioeconomic burden associated with ENTM disease.

Extrapulmonary nontuberculous mycobacteria (ENTM) disease comprises a heterogeneous group of clinical manifestations, from benign cervical lymphadenitis in children to severe skin and soft tissue infections complicated by osteomyelitis (*1*–*3*). Many ENTM cases occur from nosocomial infections and in persons without predisposing conditions (*4*,*5*). Healthcare costs associated with pulmonary nontuberculous mycobacteria are well-described (*6*,*7*), but ENTM-associated costs remain unexplored. We estimated direct and indirect costs associated with ENTM disease in Denmark.

This nationwide study included all patients >18 years of age with ENTM diagnosed during 2005–2017. We identified cases through the first occurrence of a nontuberculous mycobacteria diagnosis code (A31.1, A31.8, or A31.9) using the International Classification of Diseases, 10th Revision (ICD-10). We defined cases by using a previously described approach (*4*) (Appendix Table 1) and matched each case to 4 randomly selected comparators of the same age, sex, marital/cohabitation status, and municipality of residence in the diagnosis year. 

We considered direct healthcare costs as expenses from primary care, prescription medications, and inpatient and outpatient secondary care. We estimated indirect costs by comparing employment income between case-patients and comparators, along with public benefits (unemployment benefits, social security, sick pay, disability pensions, early retirement pensions, and age pensions) for persons 18–64 years of age. We obtained 2002–2020 patient and cost data from national health and income registries in Denmark (*8*). We applied a 2-step generalized linear model with a gamma distribution and log link to estimate annual costs for cases and comparators over the 3 years before and after ENMT diagnosis. We incorporated mortality-adjusted weights to account for differential survival. Adjusted analyses included Charlson comorbidity index (CCI) on the basis of inpatient and outpatient ICD-10 diagnosis codes, and education level (*9*). We calculated costs in 2020 euros (€). We conducted analyses using Stata 16.1 (StataCorp LLC, https://www.stata.com) and SAS 9.4 (SAS Institute, Inc., https://www.sas.com) and generated visualizations in R version 4.2.3 (The R Project for Statistical Computing, https://www.r-project.org). 

We included 406 cases and 1,580 matched comparators with a median age of 57 (IQR 30) years; of cases, 60.1% (n = 244) were among men and 39.9% (n = 162) among women (Appendix Table 2). Case-patients had lower education levels than comparators (31.3% vs. 28.6% with primary education only) and were less often employed (37.7% vs. 47.8%) (both p<0.001). Although underlying conditions were more prevalent among case-patients, most had none recorded (Appendix Table 3).

Analyses showed total direct healthcare costs for cases increased sharply around the time of diagnosis (year 0), peaking at diagnosis and remaining higher than for comparators throughout follow-up (Figure 1). Employment income was consistently lower for case-patients and declined in the 2 years preceding diagnosis. Public benefits increased substantially among case-patients, peaking around the time of diagnosis. Net costs (i.e., sum of direct costs and foregone earnings) peaked in the year before diagnosis (€21,924; 95% CI €21,017–€22,861) and at diagnosis (€20,747; 95% CI €19,960–€21,550). After adjusting for CCI and education level, direct healthcare costs were 4.3 (95% CI 3.8–4.8) times higher in cases than in comparators in the diagnosis year (Figure 2). Outpatient costs were elevated the year before diagnosis (cost ratio 4.0 [95% CI 3.6–4.5]), whereas inpatient costs peaked at diagnosis (cost ratio 6.9 [95% CI 6.2–7.7]) (Appendix Figure 1). Public benefits, specifically sick pay, disability, and early retirement pensions, rose substantially at diagnosis, and total public benefits were 1.4 (95% CI 1.2–1.6) times higher for case-patients than comparators in the diagnosis year (Appendix Figures 1, 2). Overall, ENTM cases incurred substantially higher direct and indirect costs than comparators, particularly in the peridiagnostic period. 

**Figure 1:**
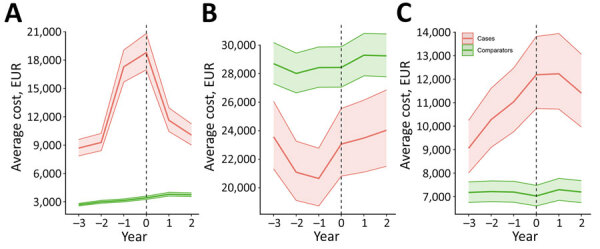
Annual average costs before and after extrapulmonary nontuberculous mycobacterial disease diagnosis, Denmark, 2005–2017. A) Total direct healthcare costs; B) employment income; C) total public benefits. Dotted vertical lines indicate year of diagnosis; solid center lines indicate average cost; shaded areas with borders indicate 95% CIs.

**Figure 2:**
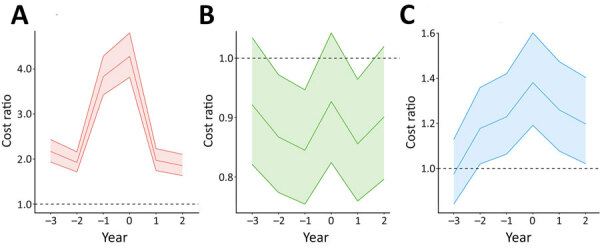
Cost ratios before and after extrapulmonary nontuberculous mycobacterial disease diagnosis, Denmark, 2005–2017. A) Total direct healthcare costs; B) employment income; C) total public benefits. Ratios adjusted for Charlson comorbidity index and educational level; values above 1 (dotted horizontal line) indicate higher costs for cases than comparators. Solid center lines indicate average cost; shaded areas with borders indicate 95% CIs.

ENTM disease is clinically heterogeneous and often requires prolonged diagnostic workup and multidisciplinary care. Rising primary care and outpatient costs before diagnosis likely reflect diagnostic delays and repeated healthcare contacts, whereas inpatient and prescription drug costs around the time of diagnosis more plausibly reflect treatment-related care. The decline in employment income and rise in public benefits before and around the time of diagnosis suggest substantial functional impairment. ENTM can occur as a device- or procedure-related infection, underscoring the need for procedural safety and infection prevention (*5*). Those infections, together with the observed cost patterns, point to opportunities for cost reduction through earlier recognition.

Although baseline socioeconomic differences might contribute to the observed cost differences, matching on key demographic variables and adjusting for education level and CCI partially addressed that concern, but we cannot exclude residual confounding. The nationwide design and registry data support the generalizability of findings to comparable tax-funded healthcare systems. However, the study was limited by a lack of species-level microbiological data and by an inability to separately quantify costs attributable to diagnostic workup, antimicrobial treatment, and surgical procedures.

In conclusion, ENTM was associated with substantial healthcare costs, reduced employment income, and increased reliance on public support in Denmark. Those findings highlight the broader socioeconomic impacts associated with ENTM before and after diagnosis. Although ENTM disease is rare (*2*,*3*), the associated costs highlight the need for early diagnosis and effective management to reduce healthcare utilization and productivity losses.

Appendix Additional information on costs of extrapulmonary nontuberculous mycobacterial disease, Denmark, 2005–2017.
