# Supplementary material for: Costs of Extrapulmonary Nontuberculous Mycobacteria Disease, Denmark, 2005–2017
Source: Emerg Infect Dis. 2026 Mar;32(3):442–4. doi: 10.3201/eid3203.251548 (PMC13016030; doi:10.3201/eid3203.251548)
Supplement: Appendix — Additional information on costs of extrapulmonary nontuberculous mycobacterial disease, Denmark, 2005–2017. [file 25-1548-Techapp-s1.pdf]

Article DOI: https://doi.org/10.3201/eid3203.251548

EID cannot ensure accessibility for supplementary materials supplied by authors. Readers who have difficulty accessing supplementary content should contact the authors for assistance.

Costs of Extrapulmonary Nontuberculous Mycobacterial Disease, Denmark, 2005–2017

Appendix

Appendix Table 1. ENTM disease case definitions by ICD-10 diagnosis and procedural codes*

ENTM diagnosis includes	ICD-10 code
At least one of the following:	
Cutaneous mycobacterial infection	A31.1
Cutaneous infection with *Mycobacterium marinum*	A31.1A
Cutaneous infection with *Mycobacterium ulcerans*	A31.1B
OR	
Other mycobacterial infections/Unspecified	A31.8 or A31.9
AND no exclusion criteria:	
Treatment by inhalation of colistin	BGHR9A
Interventions due to secretions of the airways	BGME
Endoscopy of the trachea, bronchi, and lungs	KUG
Gastric lavage	ZZ1010
Surgery of the lung	KGD
Acquiring sputum for culture	ZZ4172
Bronchiectasis	J47.9
Chronic obstructive pulmonary disease	J44
Tuberculosis	A15, A16, B90

* Based on the methodology used by Pedersen et al. Emerg Infect Dis. 2024;30:1790–1798. (https://doi.org/10.3201/eid3009.240475). ENTM, extrapulmonary nontuberculous mycobacterial; ICD-10, International Classification of Diseases, 10th Revision.

Appendix Table 2. Sociodemographic characteristics of ENTM disease cases and comparators at the index year*

Characteristics	Cases (n = 406)	Comparators (n = 1,580)	p-value†
Sex, no. (%)			Matched
F	162 (39.9)	632 (40.0)	
M	244 (60.1)	948 (60.0)	
Median age, y (IQR)	57 (30)	57 (28)	Matched
Age group, y no. (%)			Matched
18–29	35 (8.6)	134 (8.5)	
30–39	47 (11.6)	183 (11.6)	
40–49	69 (17.0)	274 (17.3)	
50–59	68 (16.7)	272 (17.2)	
60–69	70 (17.2)	276 (17.5)	
≥70	117 (28.8)	441 (27.9)	
Marital/cohabitation status, no. (%)			Matched
Single	168 (41.4)	644 (40.8)	
Married/co-living	238 (58.6)	936 (59.2)	
Region, no. (%)			Matched
North	33 (8.1)	130 (8.2)	
Central	73 (18.0)	285 (18.0)	
Southern	95 (23.4)	370 (23.4)	
Capital	133 (32.8)	514 (32.5)	
Zealand	72 (17.7)	281 (17.8)	

Characteristics	Cases (n = 406)	Comparators (n = 1,580)	p-value†
Education, no. (%)			<0.001
Primary	127 (31.3)	452 (28.6)	
Secondary	18 (4.4)	81 (5.1)	
Vocational	140 (34.5)	581 (36.8)	
Short college	17 (4.2)	61 (3.9)	
Medium college	42 (10.3)	231 (14.6)	
Master/PhD	26 (6.4)	122 (7.7)	
Unknown	36 (8.9)	52 (3.3)	
Employment status, no. (%)			<0.001
Employed	153 (37.7)	756 (47.8)	
Unemployed	34 (8.4)	89 (5.6)	
Disability pension	49 (12.1)	72 (4.6)	
Early retirement	13 (3.2)	36 (2.3)	
Age pension	140 (34.5)	545 (34.5)	
Education	8 (2.0)	49 (3.1)	
Other	9 (2.2)	33 (2.1)	
CCI score ≥1‡	152 (37.4)	186 (11.8)	<0.001

*CCI, Charlson Comorbidity Index; ENTM, extrapulmonary nontuberculous mycobacterial.
†χ^2 test case versus comparators.
Comorbidities by Charlson Comorbidity Index disease categories as defined by Quan et al. Am J Epidemiol. 2011;173:676–82 (https://doi.org/10.1093/aje/kwq433).

Appendix Table 3. Comorbidities of extrapulmonary nontuberculous mycobacterial disease cases and comparators*

Comorbidity	No. % cases, n = 406	No. % comparators, n = 1,580
Congestive heart failure	23 (5.7)	20 (1.3)
Dementia	5 (1.2)	6 (0.4)
Chronic pulmonary disease	30 (7.4)	43 (2.7)
Rheumatologic disease	35 (8.6)	14 (0.9)
Mild liver disease	0	8 (0.5)
Moderate or severe liver disease	0	0
Diabetes with chronic complications	20 (4.9)	25 (1.6)
Hemiplegia or paraplegia	0	0
Renal disease	22 (5.4)	13 (0.8)
Any malignancy, including leukemia and lymphoma	59 (14.5)	88 (5.6)
Metastatic solid tumor	11 (2.7)	0
AIDS/HIV	14 (3.4)	0
No comorbidity	254 (62.6)	1,394 (88.2)

*Comorbidities by Charlson Comorbidity Index disease categories as defined by Quan et al. Am J Epidemiol. 2011;173:676–82 (https://doi.org/10.1093/aje/kwq433).

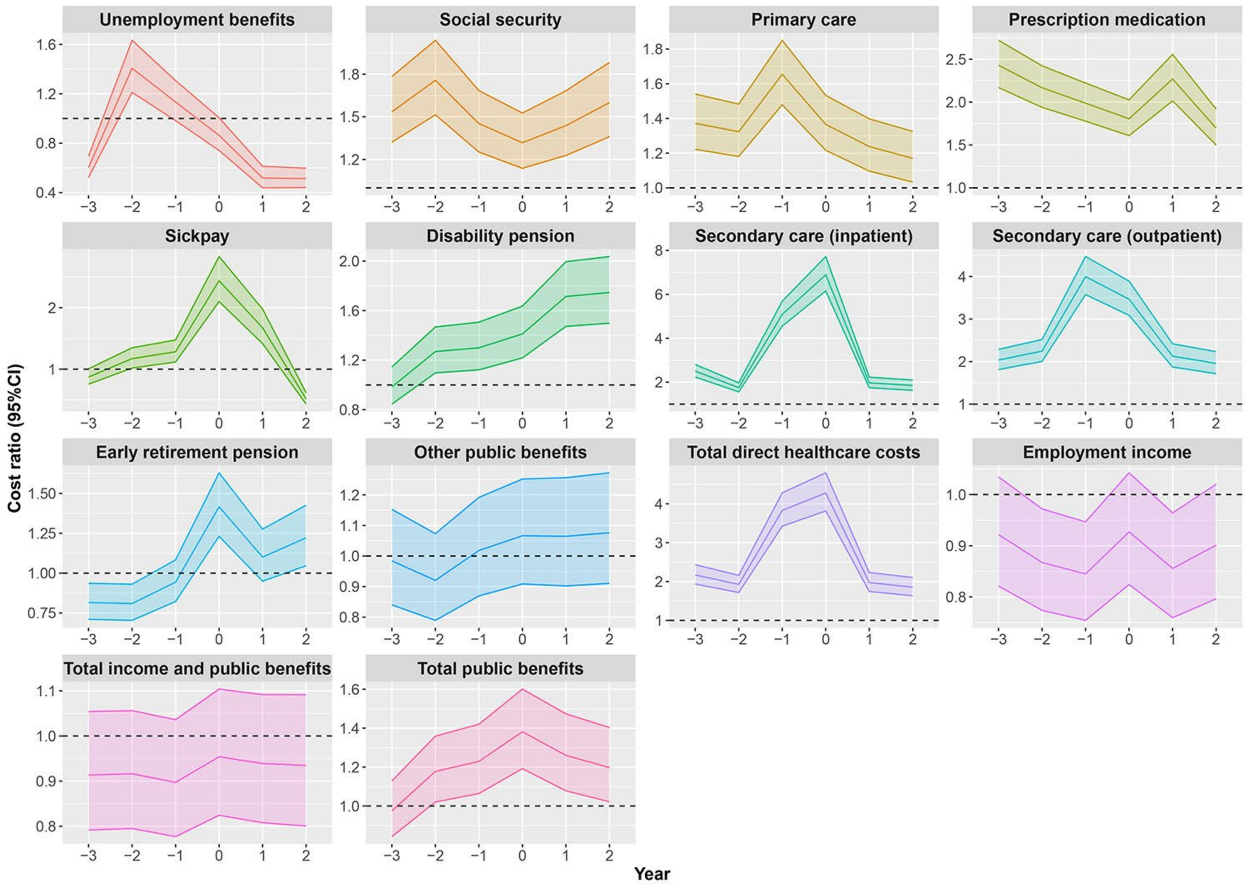

Appendix Figure 1. Cost ratios (95% CI) adjusted for Charlson Comorbidity Index and educational level three years before and after the ENTM disease diagnosis (year 0), with values above 1 indicating higher costs for cases than comparators. Abbreviations: ENTM, extrapulmonary nontuberculous mycobacterial. CI, confidence interval.

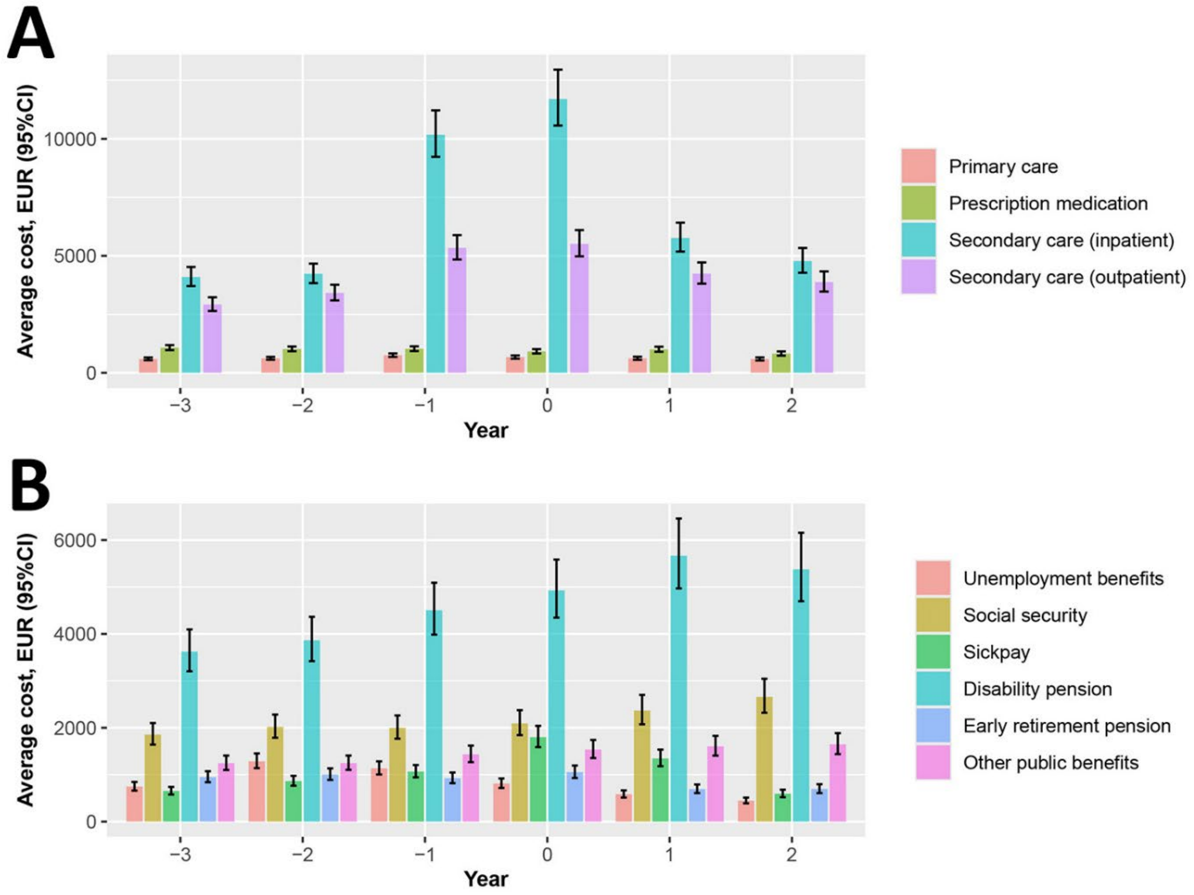

Appendix Figure 2. Annual average direct healthcare costs (i.e., primary care, prescription medications, and in- and outpatient secondary care) (panel A) and public benefits (i.e., temporary unemployment benefits and pensions*) (panel B) 3 years before and after the ENTM disease diagnosis (year 0). Abbreviations: ENTM, extrapulmonary nontuberculous mycobacterial. CI, confidence interval. *Other public benefits were defined as housing benefits, child benefits, green checks, and student grants.
